# Prognostic Value of HIFs Expression in Head and Neck Cancer: A Systematic Review

**DOI:** 10.1371/journal.pone.0075094

**Published:** 2013-09-13

**Authors:** Liang Gong, Wei Zhang, Jianding Zhou, Jie Lu, Hua Xiong, Xueli Shi, Jianqiang Chen

**Affiliations:** 1 Department of Otorhinolaryngology, Affiliated Cixi Hospital of Wenzhou Medical College, Cixi, China; 2 Department of Endocrinology, Zhejiang People's hospital, Hangzhou, China; National Taiwan University, Taiwan

## Abstract

**Background:**

Tumor hypoxia plays a fundamental role in resistance to therapy and disease progression. A number of studies have assessed the prognostic role of HIFs expression in head and neck cancer (HNC), but no consistent outcomes are reported.

**Methodology:**

A systematical search was performed to search relevant literatures in PubMed, Web of Science and ISI Web of Knowledge databases. The patients’ clinical characteristics and survival outcome were extracted. The correlation between HIFs expression and prognosis was analyzed.

**Principal Findings:**

A total of 28 studies assessed the association between HIFs and HNC survival, the result showed that overexpressed HIFs was significantly associated with increase of mortality risk (HR = 2.12; 95% CI: 1.52–2.94; I^2^ 74%). Subgroup analysis on different HIF isoforms with OS indicated that both HIF-1α and HIF-2α were associated with worse prognosis. The pooled HRs were 1.72(95% CI 1.34–2.20; I^2^ 70.7%) and 1.79(95% CI: 1.42–2.27, I^2^ 0%). Further subgroup analysis was performed by different geographical locations, disease subtype, stage, types of variate analysis and cut-off value. The results revealed that overexpressed HIF-1α was significantly associated with poor prognosis in Asian patients (HR = 2.34; 95% CI: 1.76–3.1; I^2^ 48.9%), but not in European patients (HR = 1.13; 95% CI: 0.77–1.66; I^2^ 78.3%). Furthermore, HIF-1α overexpression was significantly associated with worse OS in oral carcinoma(HR = 2.1; 95% CI: 1.11–3.97; I^2^ 81.7%), nasopharyngeal carcinoma(HR = 2.07; 95% CI:1.23–3.49; I^2^ 22.5%) and oropharynx carcinoma(HR = 1.76; 95% CI:1.05–2.97; I^2^ 61%), but not in laryngeal carcinoma(HR = 1.38; 95% CI: 0.87–2.19; I^2^ 62.5%). We also found that the prognostic value of HIF-1α overexpression existed only when using staining and percentage as positive definition (HR = 1.82; 95% CI 1.42–2.33; I^2^ 9.9%).

**Conclusions:**

This study showed that overexpressed HIFs were significantly associated with increase of mortality risk. Subgroup analysis revealed that overexpressed HIF-1α was significantly associated with worse prognosis of HNC in Asian countries. Additionally, HIF-1α had different prognostic value in different HNC disease subtypes.

## Introduction

Head and neck cancer (HNC) is the sixth most common malignance worldwide [1]. Over 70% of head and neck cancer patients present with advanced stage III and IV disease at the time of diagnosis. Despite the advance in treatment regimens including surgery, radiotherapy, chemotherapy and cetuximab, the 5-year survival rate of these patients remains only 50% [2]. It suggests that current treatments are not effective in all patients. Simultaneously, the treatments would bring about many side effects (e.g.,swallowing problems, hearing loss, mucositis, late toxicity). Therefore, it is urgent to identify reliable outcome predictors in this setting.

Tumor hypoxia serve as a prognostic factor associated with worse outcome in most solid tumors, including HNC. Hypoxia has also been recognized as a major cause of failure of radiotherapy and of chemotherapy with radiomimetic drugs (i.e.,bleomycin) in HNC patients [3], [4]. For this reason, it is especially important to measure tumor oxygen levels to identify patients who would respond best to radiation or to bleomycin-containing regimens. The current ‘gold standard’ to measure tumor oxygen levels is using direct polarographic measurements, but it is invasive and not suitable for all situations. Increasingly, evaluation of hypoxia in the clinic is shifting to the monitoring of endogenous markers. The hypoxia­inducible factors (HIFs) are the best characterized markers mediating cellular responses to hypoxic stress. Of the three HIF family members, HIF-1 and HIF-2 are the most well-characterized.

Although a number of studies have shown high tumor expression of HIF-1 and HIF-2 as adverse prognostic features in HNC[5]–[7], it has not been a universal finding [8], [9]. Therefore, it is necessary to analyze the data of HIFs systematically in HNC to draw a reasonable conclusion about its prognostic significance.

## Materials and Methods

### Identification and Eligibility of Relevant Studies

Literature searching was conducted from PubMed, Web of Science and ISI Web of Knowledge databases using the terms: “HIF”, “neoplasms”, “cancer”, “tumor”, “head and neck”, “oral”, “pharyngeal”, “oropharyngeal”, “hypopharyngea”, “maxillofacial”, “laryngeal”, “paranasalsinus”, “prognosis” with all possible combinations. We also performed a manual search of the references of all identified articles for additional eligible studies.

The inclusion criteria for eligibility of a study in the meta-analysis were as follows: (1) evaluating HIFs expression in the human HNC tissues; (2) investigating the relationships between HIFs with prognosis; (3) providing sufficient data to estimate hazard ratio (HR) about overall survival (OS) or disease free survival (DFS). In addition, letters, reviews, conference abstracts, case reports or experiment on animal models were not in the scope of our analysis. Overlapping articles were also excluded from this meta-analysis, only the most recent or the most complete study was involved in the analysis.

### Data Extraction and Management

Two investigators (L.G. and W.Z.) reviewed each eligible study independently and extracted data from all the publications meeting the inclusion criteria. Controversial problems were resolved by discussion amongst the team of pathologists. Information was carefully retrieved from each study, using a standardized data collection form, including the following items: the first author’s name, year of publication, country of origin, number, gender and age of patients, disease subtype, stage, follow-up, survival data, HIF isoforms, treatment and cut-off value.

### Methodological Assessment

The methodological quality of the included studies was assessed using the Newcastle–Ottawa quality assessment scale (NOS) [10]. A total of 0 and 9-star were respectively designated as lowest and highest quality, and the studies with 6-star or more were graded as the high quality ones in the scale. The scores provided by two researchers were compared and a consensus value for each item was achieved.

### Statistical Methods

For the pooled analysis of the impact of HIFs expression on survival outcome, HRs and its 95% CI were used. But these statistical variables were not described explicitly in most studies. Therefore, we calculated the necessary statistics from available numerical data in the articles according to the methods described by Parmar, if the studies offered the data such as log-rank test p values, number of total events, the number of aberrant HIFs expression and number of preserved HIFs expression. If not available, we extracted time-to-event data from the Kaplan–Meier curves for HR and its 95% CI calculation. Kaplan-Meier curves were read by Engauge Digitizer version4.1 (http://digitizer.sourceforge.net/). The impact of overexpressed/low HIF expression on survival was considered to be statistically significant if the 95% CI did not overlap with 1. The effect of between-study heterogeneity in this meta-analyses was assessed by Chi- square based Q statistical test [12]. And the I^2^ statistic was used to quantify the proportion of the total variation [13]. When a significant heterogeneity existed across the included studies (I^2^>50%), the random effects model (the DerSimonian and Laird method) was used for meta-analysis [14]. Otherwise, we chose the fixed-effects model when the studies were found to be homogeneous (I^2^>50%) [15]. The possibility of publication bias was assessed by visually assessing the symmetry of Begg’s funnel plots and then was further quantitatively performing Egger’s test [16]. Publication bias was indicated when p value of Egger’s test <0.05. The meta-analysis was performed using STATA version 12.0 software (Stata Corporation, Collage Station, Texas, USA). A two-sided P value less than 0.05 was considered statistically significant. Sensitivity analyses were performed by excluding each study individually to evaluate stability of the results.

## Results

### Characteristics of Studies

**Figure 1:**
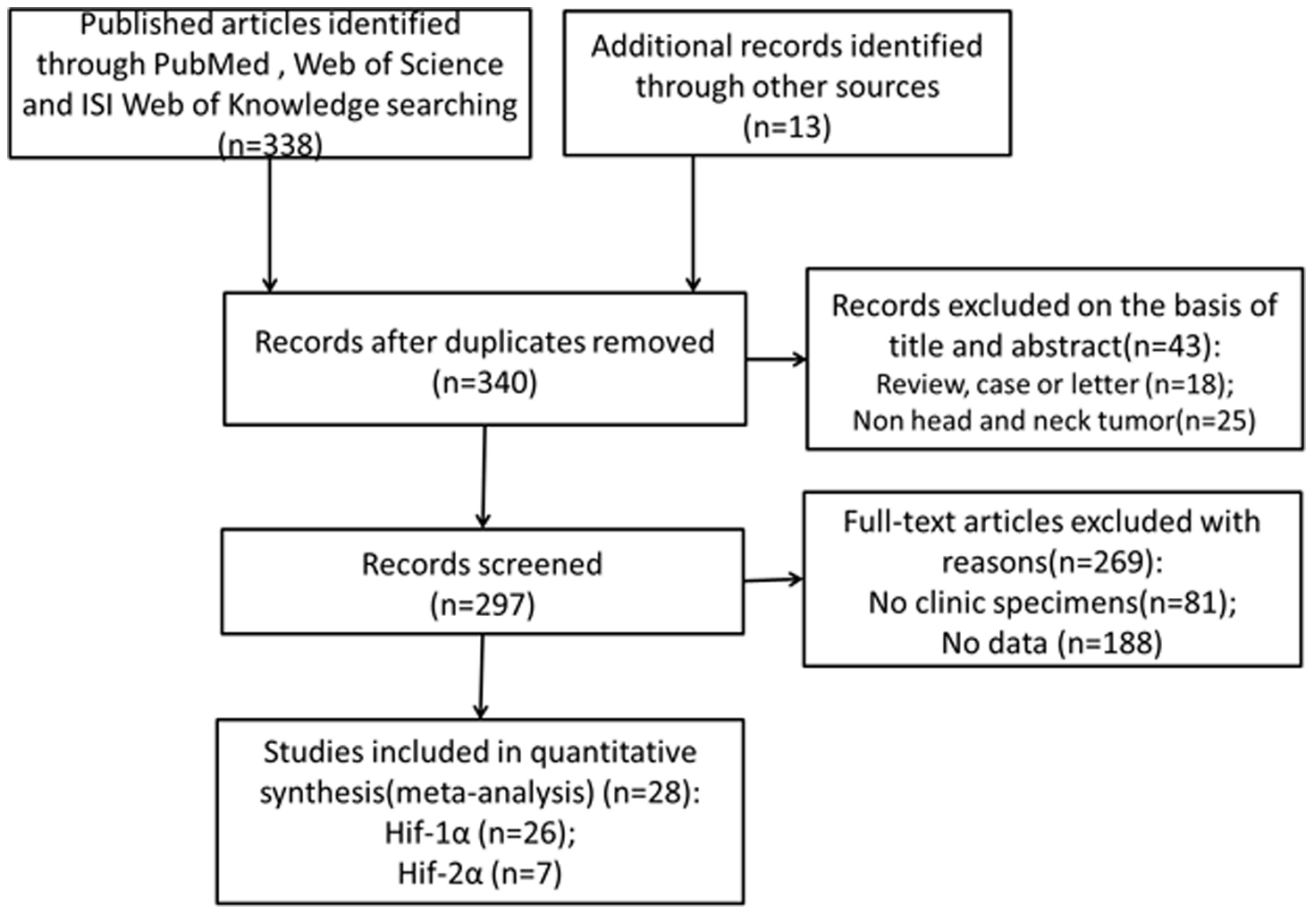
Flow diagram of study selection procedure.

As shown in **Figure 1**, initial search retrieved 351 published studies. After exclusion of the studies that did not meet the inclusion criteria, a total of 28 eligible studies were included in the final meta-analysis[5]–[9], [17]–[38]. Of these 28 publications, 26 studies assessed the relationships between HIF-1α expression with HNC prognosis[5]–[9], [17]–[22], [24]–[38], while 7 studies evaluated the association of HIF-2α expression and prognosis[7], [8], [21]–[23], [38]. The clinical features of these 28 included studies were summarized in **Table S1**. A total of 2293 HNC patients were enrolled in this meta-analysis and the included studies were published from 2002 to 2013. Sample sizes ranged from 21 to 233 patients (mean 81). The median age of patients ranged from 48 to 66 years old. 21 of these studies enrolled less than 100 patients and 7 studies included more than 100 patients. 17 of these studies evaluated patients from Asia, 11 evaluated the patients from Europe or Australia.

### Impact of HIFs Expression on Overall Survival of Head and Neck Cancer

**Figure 2:**
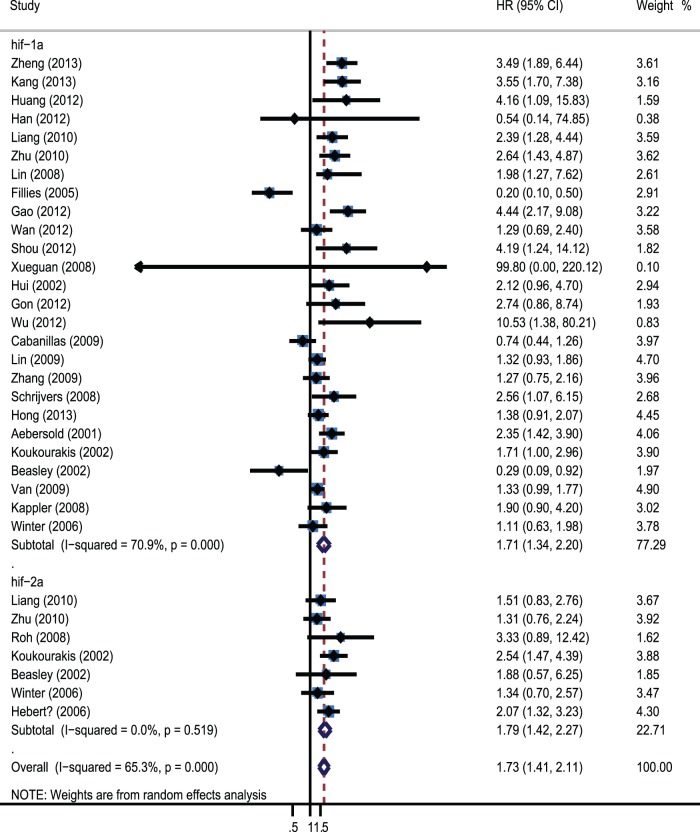
Forrest plot of Hazard ratio for the association of different HIF isoforms expression with overall survival.

A forest plot of the individual HR estimates and results from the meta-analysis are presented in **Figure 2**. Overall, overexpressed HIFs were significantly associated with increase of mortality risk. The pooled HR was 2.12(95% CI 1.52–2.94; I^2^ 74%) in the random effects model. We also performed subgroup analysis about association of different HIF isoforms with OS, the results showed that both HIF-1α and HIF-2α were associated with worse prognosis. The pooled HRs were 1.72(95% CI 1.34–2.20; I^2^ 70.7%) and 1.79(95% CI: 1.42–2.27, I^2^ 0%).

### Subgroup Analyses

**Figure 3:**
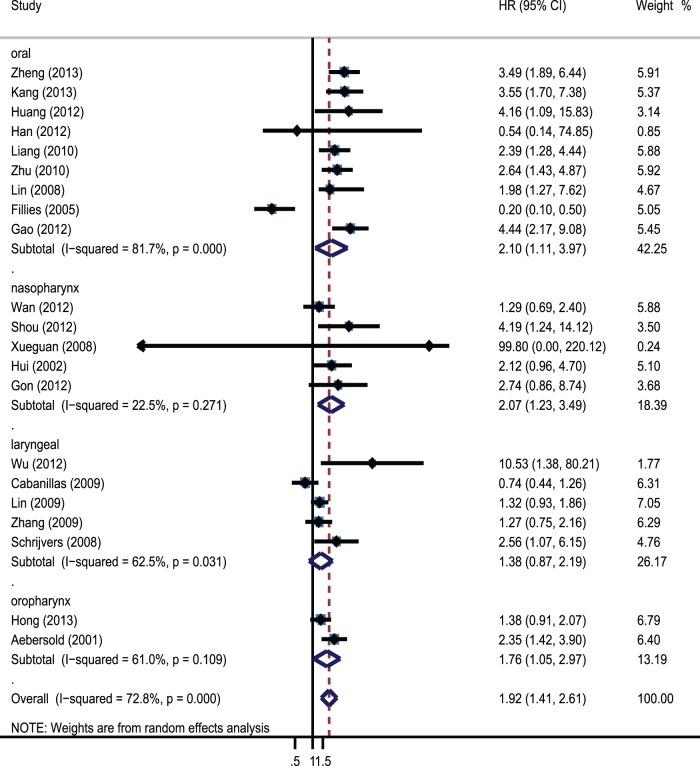
Forrest plot of Hazard ratio for the association of overexpressed Hif-1α with different disease subtype.

**Figure 4:**
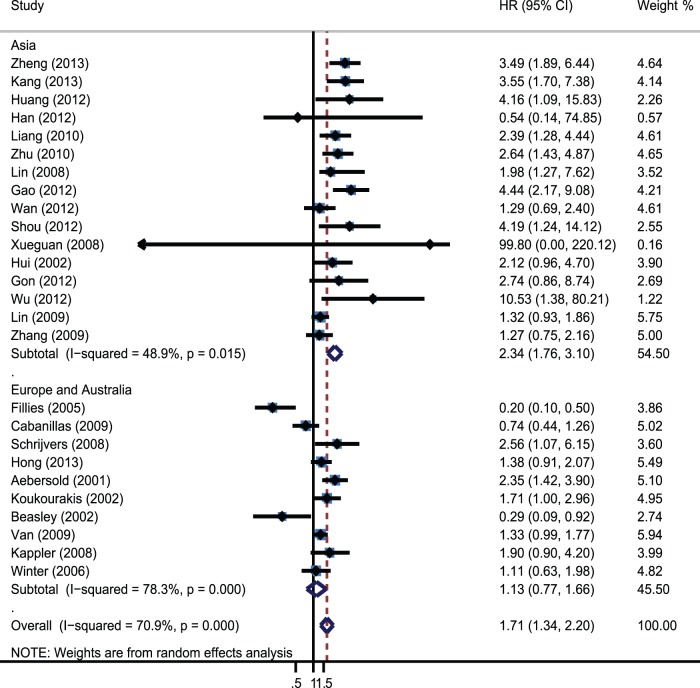
Forrest plot of Hazard ratio for the association of overexpressed Hif-1α with different geographical locations.

Moreover, further subgroup analysis was performed to investigate the prognostic impact of HIF-1α and HIF-2α in HNC patients with different disease subtype, geographical locations, stage, types of variate analysis and cut-off value. Subgroup analysis indicated HIF-1α overexpression was significantly associated with worse OS in oral carcinoma (HR = 2.1; 95% CI: 1.11–3.97; I^2^ 81.7%), nasopharyngeal carcinoma (HR = 2.07; 95% CI:1.23–3.49; I^2^ 22.5%) and oropharynx carcinoma(HR = 1.76; 95% CI:1.05–2.97; I^2^ 61%), but not in laryngeal carcinoma(HR = 1.38; 95% CI: 0.87–2.19; I^2^ 62.5%). The pooled HRs were 2.1(95% CI 1.11–3.97; I^2^ 81.7%), 2.07(95% CI 1.23–3.49; I^2^ 22.5%), 1.76(95% CI 1.05–2.97; I^2^ 61%) and 1.38(95% CI 0.87–2.19; I^2^ 74%), respectively **(** **Figure 3** **)**. Furthermore, a significant relation between HIF-1α overexpression and OS was exhibited in Asian patients (HR = 2.34; 95% CI: 1.76–3.1; I^2^ 48.9%) **(** **Figure 4** **)**, in studies with II-IV patients (HR = 2.07; 95% CI 1.19–3.59; I^2^ 0%) and using staining and percentage as positive definition (HR = 1.82; 95% CI 1.42–2.33; I^2^ 9.9%). Other factors comprising method of variate analysis, and distribution of HIF-1α did not alter the significant OS of overexpressed HIF-1α **(** **Table 1** **)**. On the other hand, overexpressed HIF-2α in the nucleus and cytoplasm was significantly correlated with a worse prognosis (HR = 2.04; 95% CI: 1.51–2.75; I^2^ 0%).

**Table 1:** Stratified analysis of pooled hazard ratios for head and neck cancer with overexpressed Hif-1α and Hif-2α.

	Hif-1α						Hif-2α						
					Heterogeneity						Heterogeneity		
	Number of studies	Number of patients	Pooled HR(95% CI)	P value	I^2^(%)	P value	Number of studies	Number of patients	Pooled HR(95% CI)	P value	I^2^(%)	P value	
Disease subtype													
Head and neck (total)	26	2293	1.71(1.34.-2.2)	0	70.90%	0	7	710	1,79(1.42–2.27)	0	0%	0.519	
Nasopharynx	5	445	2.07(1.23–3.49)	0.006	22.50%	0.271	–	–	–	–	–	–	
Laryngeal	5	458	1.38(0.87–2.19)	0.168	62.50%	0.031	–	–	–	–	–	–	
Oropharynx	2	331	1.76(1.05–2.97)	0.033	61%	0.109	–	–	–	–	–	–	
Oral	9	650	2.1(1.11–3.97)	0.023	81.70%	0	3	207	1.5(1.02–2.2)	0.038	0%	0.437	
Study location													
Europe and Australia	10	1013	1.13(0.77–1.66)	0.521	78.30%	0	4	503	2(1.48–2.68)	0	0%	0.529	
Asia	16	1280	2.34(1.76–3.1)	0	48.90%	0.015	3	207	1.5(1.02–2.2)	0.038	0%	0.437	
Definition of positive													
Staining	4	345	1.98(0.85–4.58)	0.112	80.80%	0.001	–	–	–	–	–	–	
Percentage	12	1034	1.46(0.93–2.28)	0.098	76.80%	0	2	100	2.44(1–5.92)	0.049	0%	0.531	
Staining and percentage	8	1108	1.82(1.42–2.33)	0	9.90%	0.353	4	513	1.89(1.44–2.48)	0	0%	0.412	
Disease Stage													
I-IV	10	879	1.59(0.96–2.62)	0.069	81.40%	0	2	118	1.49(0.91–2.46)	0.115	39.50%	0.198	
II-IV	3	183	2.07(1.19–3.59)	0.01	0%	0.455	–	–	–	–	–	–	
III-IV	2	235	1.32(1.02–1.72)	0.037	0%	0.93	–	–	–	–	–	–	
Variate analysis													
Multivariate	15	1378	1.68(1.21–2.32)	0.002	73.70%	0	2	349	1.8(1.25–2.6)	0.002	14.20%	0.28	
Univariate	11	915	1.78(1.17–2.7)	0.007	69.10%	0	5	361	1.79(1.32–2.43)	0	0.70%	0.402	
Positive site													
Nuclear	16	1499	1.52(1.03–2.25)	0.037	77.50%	0	3	327	1.37(0.93–2.03)	0.115	0%	0.859	
Nuclear/cytoplasm	8	654	1.98(1.44–2.74)	0	46.90%	0.068	3	362	2.04(1.51–2.75)	0	0%	0.457	
Quality score													
≥6	18	1614	1.97(1.49–2.61)	0	66.50%	0	4	340	1.73(1.27–2.36)	0.001	4.40%	0.371	
<6	8	679	1.22(0.74–2.03)	0.432	74.30%	0	3	370	1.88(1.32–2.68)	0	0%	0.379	

### Sensitivity Analyses and Publication Bias

Sensitivity analysis indicated that the pooled HRs were not significantly inﬂuenced by omitting any single study **(Table S2)**. Egger’s test indicated that there was no evidence of significant publication bias after assessing the funnel plot for the studies included in our meta-analysis (**Figure S1–S2**).

## Discussion

Hypoxia is a feature of most tumors and it often arises because of rapid cell division and aberrant tumor angiogenesis and blood flow. Persistent hypoxia leads to a selection of genotypes favoring survival and promoting tumor angiogenesis, epithelial-to-mesenchymal transition, invasiveness and metastasis, as well as suppressing immune reactivity [39]. Owing to these effects on tumour development, hypoxia is suggested to be a prognostic factor associated with resistance to therapy and disease progression in various types of cancer, including HNC.

A key molecular mediator of the cellular responses to hypoxia is the HIFs (HIF-1/2α). Under aerobic conditions, HIF-1/2α is hydroxylated by specific prolyl hydroxylases (PHDs), which facilitates binding of von Hippel–Lindau protein (pVHL) to the HIF-1/2α. pVHL forms the substrate recognition module of an E3 ubiquitin ligase complex. Then, HIF-1/2α protein is rapidly and continuously degraded by ubiquitination and proteasomal degradation. However, under hypoxic conditions, prolylhydroxylases become inactivated, and thus, HIF-1/2α is stabilized and activated. Subsequently, it would activate the expression of downstream target genes that regulate several biological processes including angiogenesis, cell proliferation and survival, glucose metabolism, pH regulation and migration. There is a growing body of evidence that points to a fundamental role of HIFs in tumor progression. But some changes in hypoxic cells can also result in increased drug sensitivity [40]; these exceptions caution against that hypoxic cells are invariably chemoresistant. In addition, there is no consensus on the association between high expression of HIFs and adverse prognostic features in HNC at present. Thus, we performed a quantitative meta-analysis to determine the association between HIFs expression and the prognosis of HNC.

In this meta-analysis, 28 studies assessed the association between HIFs and HNC survival, the results showed that overexpressed HIFs were significantly associated with increase of mortality risk (HR = 2.12; 95% CI: 1.52–2.94; I^2^ 74%). Subgroup analysis on different HIF isoforms with OS indicated that both HIF-1α and HIF-2α were associated with worse prognosis. The pooled HRs were 1.72(95% CI 1.34–2.20; I^2^ 70.7%) and 1.79(95% CI: 1.42–2.27, I^2^ 0%). Further subgroup analysis was performed by different geographical locations, disease subtype, stage, types of variate analysis and cut-off value. The results revealed that overexpressed HIF-1α was only significantly associated with poor prognosis in Asian countries, while not in European countries. It suggested that HIF-1α overexpression could be racial different as a prognostic factor. Furthermore, HIF-1α overexpression was significantly associated with worse OS in oral carcinoma, nasopharyngeal carcinoma and oropharynx carcinoma, but not in laryngeal carcinoma (HR = 1.38; 95% CI: 0.87–2.19; I^2^ 74%). This result suggested that HIF-1α had distinct prognostic significance in different HNC disease types. In addition, we found that and a positive definition of HIF-1α also altered the prognostic significance. The prognostic value of HIF-1α overexpression existed only when using staining and percentage (HR = 1.82; 95% CI 1.42–2.33; I^2^ 9.9%). It proposed that we needed to combine staining and percentage methods to evaluate the expression of HIF-1α in clinic diagnosis. Moreover, significant correlations were also observed when the methodological quality of studies was ≥6, the significance disappeared when <6. In this meta-analysis, we had dealt with highly significant heterogeneity among the 26 studies assessing the association between HIF-1α and OS. Subgroup analysis suggested that the heterogeneity in these studies could be partially explained by disease subtype, definition of positive and disease stage. When the analysis of OS was performed without consideration of these other factors, heterogeneity was detected (I^2^ 70.9% P = 0.000). When the analysis was limited to studies of nasopharynx and oropharynx, heterogeneity was not detected. Heterogeneity was also not detected when the analysis was limited to studies that defined HIF-1α positive using staining and percentage methods and the studies of II-IV and III-IV patients. Sensitivity analyses were performed to ensure that the results were reliable and valid.

However, there were several limitations to be considered in this meta-analysis. First, sample size of the total patients was limited and only 7 included studies were >100 in our meta-analysis, the mean sample size was relative small with 89.7 (ranging from 21 to 233). The relative small size of the sample can inevitably increase the risk of bias in this meta-analysis. Second, certain reports with negative or controversial results might not be reported, and therefore, publication biasis were inevitable.

In conclusion, the findings from this systematic review suggested that overexpressed HIFs were significantly associated with increase of mortality risk. Subgroup analysis revealed that overexpressed HIF-1α was significantly associated with worse prognosis of HNC in Asian countries. Additionally, HIF-1α overexpression was significantly associated with worse OS in oral carcinoma, nasopharyngeal carcinoma and oropharynx carcinoma, but not in laryngeal carcinoma. However, more well designed and large sample size studies are needed to provide further evidence.

## Supporting Information

Figure S1 **Egger’s Publication bias plot for studies regarding the association of Hif-1α expression with overall survival: the relationship between the effect size of individual studies (HR, vertical axis) and the precision of the study estimate (standard error, horizontal axis).**
(TIF)

Figure S2 **Egger’s Publication bias plot for studies regarding the association of Hif-2α expression with overall survival.**
(TIF)

Table S1 **Characteristics of studies included in the meta-analysis.**
(DOCX)

Table S2 **HRs (95% CI) of sensitivity analysis for the meta-analysis.**
(DOCX)

Table S3 **PRISMA checklist.**
(DOC)
