# Supplementary figures and images for: Prognostic Value of HIFs Expression in Head and Neck Cancer: A Systematic Review
Source: PLoS One. 2013 Sep 13;8(9):e75094. doi: 10.1371/journal.pone.0075094 (PMC3772872; doi:10.1371/journal.pone.0075094)

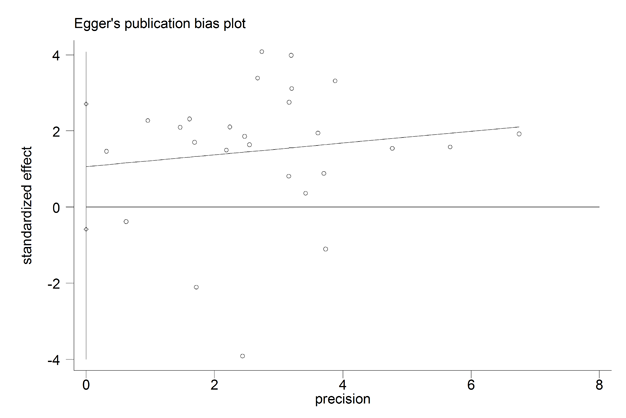

Supplement: Figure S1 — Egger’s Publication bias plot for studies regarding the association of Hif-1α expression with overall survival: the relationship between the effect size of individual studies (HR, vertical axis) and the precision of the study estimate (standard error, horizontal axis). (TIF) [file pone.0075094.s001.tif]

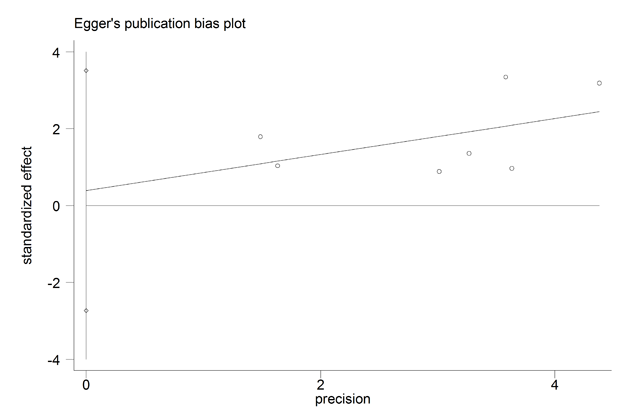

Supplement: Figure S2 — Egger’s Publication bias plot for studies regarding the association of Hif-2α expression with overall survival. (TIF) [file pone.0075094.s002.tif]
