# Supplementary material for: Prognostic Value of HIFs Expression in Head and Neck Cancer: A Systematic Review
Source: PLoS One. 2013 Sep 13;8(9):e75094. doi: 10.1371/journal.pone.0075094 (PMC3772872; doi:10.1371/journal.pone.0075094)
Supplement: Table S1 — Characteristics of studies included in the meta-analysis. (DOCX) [file pone.0075094.s003.docx]

**Table S1. Characteristics of studies included in the meta-analysis.**

| **First**  **author** | **Year** | **Country** | **Patient**  **(M/F)** | **Age**  **(year)** | **Tumor**  **site** | **Positive**  **site** | [**Pathological**](app:ds:pathological) [**type**](app:ds:type) | **Stage** | **Follow-up**  **Time(month)** |
| --- | --- | --- | --- | --- | --- | --- | --- | --- | --- |
| Zheng | 2013 | China | 120(66/54) | 57mean | Oral | N | NA | I-IV | 47median |
| Kang | 2013 | China | 49(28/21) | 69.2mean | Oral | N | Squamous carcinoma | NA | NA |
| Huang | 2012 | China | 80(46/34) | NA | Oral | N/C | Squamous carcinoma | I-IV | 69median |
| Han | 2012 | Korea | 33(20/13) | 54mean | Oral | N | Squamous carcinoma | NA | 40mean |
| Liang | 2010 | China | 89(19/40) | NA | Oral | N/C | Squamous carcinoma | NA | 45median |
| Zhu | 2010 | China | 97(52/45) | 58median | Oral | N | Squamous carcinoma | I-IV | 47median |
| Roh | 2008 | Korea | 21(18/3) | 64median | Oral | NA | Salivary duct carcinoma | I-IV | 55median |
| Lin | 2008 | China | 57(54/3) | 49mean | Oral | N | Squamous carcinoma | I-IV | NA |
| Fillies | 2005 | England | 55(26/29) | 58median | Oral | N | Squamous carcinoma | I-IV | 51.8mean |
| Gao | 2012 | China | 49(28/21) | 69.2mean | Oral | N/C | Squamous carcinoma | NA | NA |
| Wan | 2012 | China | 144(113/31) | NA | Nasopharynx | N/C | NA | III-IV | NA |
| Shou | 2012 | China | 60(42/18) | 48median | Nasopharynx | N | Nonkeratinizing carcinoma | I-IV | 66median |
| Xueguan | 2008 | China | 59(48/11) | 48median | Nasopharynx | N | Squamous carcinoma | II-IV | 63median |
| Hui | 2002 | China | 90(73/17) | 45median | Nasopharynx | N | Squamous carcinoma  and undifferentiated carcinoma | II-IV | 49.56median |
| Gong | 2012 | China | 92(74/18) | 64median | Nasopharynx | N/C | Squamous carcinoma | I-IV | NA |
| Wu | 2012 | China | 49(43/6) | 60.8mean | Laryngeal | C | Squamous carcinoma  and adenoid cystic carcinoma | NA | 42.6mean |
| Cabanillas | 2009 | Australia | 106(NA) | NA | Laryngeal | N | Squamous carcinoma | I-IV | 47.5median |
| Lin | 2009 | China | 91(67/24) | 60median | Laryngeal | N/C | Squamous carcinoma | I-IV | 91median |
| Zhang | 2009 | China | 121(99/22) | 59mean | Laryngeal | N | Squamous carcinoma | I-IV | 51.5median |
| Schrijvers | 2008 | Holland | 91(81/10) | 66median | Laryngeal | N | Squamous carcinoma | NA | 40median |
| Hong | 2013 | Australia | 233(186/47) | 58.2mean | Oropharynx | N | Squamous carcinoma | NA | 51median |
| Aebersold | 2001 | Switzerland | 98(NA) | 57median | Oropharynx | N | Squamous carcinoma | NA | 31.2median |
| Koukourakis | 2002 | Greece | 75(NA) | NA | Head and neck | N/C | Squamous carcinoma | NA | 48median |
| Beasley | 2002 | England | 79(54/25) | 62median | Head and neck | N | Squamous carcinoma | NA | NA |
| Van | 2009 | Holland | 91(70/21) | 54median | Head and neck | N/M | Squamous carcinoma | III-IV | 18median |
| Kappler | 2008 | Germany | 34(29/5) | 59median | Head and neck | N/C | NA | II-IV | 27median |
| Winter | 2006 | England | 151(107/44) | NA | Head and neck | N | Squamous carcinoma | NA | 33mean |
| Koukourakis | 2006 | England | 198(NA) | NA | Head and neck | N/C | Squamous carcinoma | NA | NA |

| **First**  **author** | **Treatment**  **protocol** | **HIFs**  **isoforms** | **Definition of HIFs**  **positive** | **HR of OS**  **(95%CI)** | **Multivariate analysis** | **Quality**  **Score** |
| --- | --- | --- | --- | --- | --- | --- |
| Zheng | Surgery | Hif-1α | Nuclear staining | 3.489(1.899-6.444) | Univariate | 7 |
| Kang | Surgery | Hif-1α | Moderate/strong staining | 3.545(1.704-7.377) | Multivariate | 7 |
| Huang | Surgery | Hif-1α | Sum the intensity and percentage scores ≥4 | 4.16(1.093-15.83) | Multivariate | 5 |
| Han | Surgery/surgery+postoperative RT | Hif-1α | 10% | 0.54(0.14-74.85) | Multivariate | 4 |
| Liang | Surgery | Hif-1α | 25% and strong staining | 2.389(1.285-4.443) | Univariate | 7 |
|  |  | Hif-2α | 25% and strong staining | 1.514(0.831-2.757) | Univariate |  |
| Zhu | Surgery/surgery+postoperative RT or chemotherapy | Hif-1α | Nuclear staining | 2.641(1.433-4.868) | Multivariate | 8 |
|  |  | Hif-2α | Nuclear staining | 1.306(0.762-2.241) | Univariate |  |
| Roh | Surgery/surgery+postoperative RT | Hif-2α | Multiply the intensity and percentage scores >median score | 3.325(0.89-12.425) | Univariate | 4 |
| Lin | Surgery/surgery+postoperative RT | Hif-1α | 60% | 1.978(1.271-7.621) | Multivariate | 5 |
| Fillies | Surgery/surgery+postoperative RT | Hif-1α | 10% | 0.2(0.1-0.5) | Multivariate | 4 |
| Gao | Surgery | Hif-1α | 10% | 4.437(2.169-9.077) | Multivariate | 6 |
| Wan | IC/CRT or IC/RT | Hif-1α | Multiply the intensity and percentage scores ≥5 | 1.29(0.693-2.401) | Multivariate | 7 |
| Shou | RT with or without chemotherapy | Hif-1α | NA | 4.192(1.244-14.124) | Univariate | 7 |
| Xueguan | RT plus carbogen and nicotinamide | Hif-1α | 10% | 99.8(0.001-220.12) | Multivariate | 7 |
| Hui | Chemoradiotherapy or RT | Hif-1α | 5% | 2.12(0.96-4.7) | Univariate | 6 |
| Gong | NA | Hif-1α | Sum the intensity and percentage scores >2 | 2.737(0.857-8.742) | Univariate | 7 |
| Wu | NA | Hif-1α | 10% | 10.529(1.382-80.208) | Univariate | 6 |
| Cabanillas | Surgery/surgery+postoperative RT | Hif-1α | 10% | 0.744(0.44-1.258) | Univariate | 6 |
| Lin | NA | Hif-1α | 30% | 1.319(0.934-1.863) | Multivariate | 7 |
| Zhang | NA | Hif-1α | 10% | 1.269(0.751-2.164) | Univariate | 5 |
| Schrijvers | RT | Hif-1α | 0.50% | 2.56(1.07-6.15) | Multivariate | 4 |
| Hong | RT or RT+chemotherapy or surgery+/-RT+/-chemotherapy | Hif-1α | 10% | 1.38(0.91-2.07) | Multivariate | 7 |
| Aebersold | RT/RT+chemotherapy | Hif-1α | 10% and moderate or marked staining | 2.35(1.42-3.9) | Multivariate | 6 |
| Koukourakis | RT+chemotherapy | Hif-1α | Nuclear staining and/or 36% cytoplasmic staining | 1.711(1.001-2.962) | Univariate | 6 |
|  |  | Hif-2α | Nuclear staining and/or 23% cytoplasmic staining | 2.538(1.468-4.386) | Univariate |  |
| Beasley | Surgery/surgery+postoperative RT | Hif-1α | Any expression | 0.292(0.093-0.915) | Univariate | 6 |
|  |  | Hif-2α | Any expression | 1.882(0.567-6.25) | Univariate |  |
| Van | Chemoradiotherapy | Hif-1α | Continuous variable | 1.33(0.99-1.77) | Multivariate | 4 |
| Kappler | Chemoradiotherapy | Hif-1α | Multiply the intensity and percentage scores >1 | 1.9(0.9-4.2) | Univariate | 7 |
| Winter | Surgery/surgery+postoperative RT | Hif-1α | Median value | 1.11(0.63-1.98) | Multivariate | 5 |
|  |  | Hif-2α | Median value | 1.34(0.7-2.57) | Multivariate |  |
| Koukourakis | RT | Hif-2α | Nuclear staining>10% or strong cytoplasmic staining>50% | 2.07(1.32-3.23) | Multivariate | 5 |

M/F, male/female; NA, unavailable; N, nuclear; C, cytoplasm; N/C, nuclear/cytoplasm; N/M, nuclear/membrane; RT, radiotherapy; IC/RT, induction chemotherapy and radiotherapy; IC/CRT, induction chemotherapy plus concurrent chemoradiotherapy; Hifs, hypoxia-inducible factors; HR, hazard ratio; OS, overall survival; 95%CI, 95% confidence interval.
