# Supplementary material for: Prognostic Value of HIFs Expression in Head and Neck Cancer: A Systematic Review
Source: PLoS One. 2013 Sep 13;8(9):e75094. doi: 10.1371/journal.pone.0075094 (PMC3772872; doi:10.1371/journal.pone.0075094)
Supplement: Table S2 — HRs (95% CI) of sensitivity analysis for the meta-analysis. (DOCX) [file pone.0075094.s004.docx]

**Table S2. HRs (95% CI) of sensitivity analysis for the meta-analysis.**

**1. HIF-1α versus OS**

| **Study omitted** | **Estimated HR** | **low value**  **of 95%CI** | **High value**  **of 95%CI** |
| --- | --- | --- | --- |
| Zheng (2013) | 1.6526233 | 1.2862843 | 2.1232972 |
| Kang (2013) | 1.6589818 | 1.2898678 | 2.133723 |
| Huang (2012) | 1.6783876 | 1.3052324 | 2.1582248 |
| Han (2012) | 1.7254542 | 1.3425457 | 2.2175725 |
| Liang (2010) | 1.6875201 | 1.3039163 | 2.1839778 |
| Zhu (2010) | 1.6783007 | 1.2986208 | 2.1689885 |
| Lin (2008) | 1.7059516 | 1.319699 | 2.2052534 |
| Fillies (2005) | 1.8349637 | 1.4777526 | 2.278522 |
| Gao (2012) | 1.6392783 | 1.2809652 | 2.0978191 |
| Wan (2012) | 1.7395554 | 1.3413777 | 2.2559288 |
| Shou (2012) | 1.6733614 | 1.3015159 | 2.151444 |
| Xueguan (2008) | 1.7018681 | 1.3274627 | 2.1818728 |
| Hui (2002) | 1.7002045 | 1.3145033 | 2.1990781 |
| Gon (2012) | 1.691967 | 1.3124886 | 2.1811638 |
| Wu (2012) | 1.6744986 | 1.3070401 | 2.1452634 |
| Cabanillas (2009) | 1.7879537 | 1.3922774 | 2.2960789 |
| Lin (2009) | 1.7465056 | 1.3351319 | 2.2846296 |
| Zhang (2009) | 1.7436469 | 1.342409 | 2.2648122 |
| Schrijvers (2008) | 1.6887283 | 1.307945 | 2.1803694 |
| Hong (2013) | 1.7397734 | 1.3335409 | 2.2697554 |
| Aebersold (2001) | 1.686484 | 1.3012458 | 2.1857731 |
| Koukourakis (2002) | 1.7168891 | 1.3211319 | 2.2311988 |
| Beasley (2002) | 1.7926068 | 1.4060932 | 2.285367 |
| Van (2009) | 1.7481319 | 1.3310801 | 2.2958536 |
| Kappler (2008) | 1.7079394 | 1.3195121 | 2.2107086 |
| Winter (2006) | 1.7534269 | 1.3529427 | 2.2724583 |
| Combined | 1.71344 | 1.3353971 | 2.1985044 |

| **Study omitted** | **Estimated HR** | **low value of 95%CI** | **High value of 95%CI** |
| --- | --- | --- | --- |
| Liang (2010) | 1.8508109 | 1.4345535 | 2.3878515 |
| Zhu (2010) | 1.9328612 | 1.4897753 | 2.5077288 |
| Roh (2008) | 1.7590235 | 1.3860735 | 2.2323232 |
| Koukourakis (2002) | 1.6602997 | 1.280802 | 2.1522412 |
| Beasley (2002) | 1.7914497 | 1.4104837 | 2.2753129 |
| Winter (2006) | 1.8749526 | 1.4581784 | 2.4108486 |
| Hebert (2006) | 1.7004572 | 1.2912058 | 2.2394223 |
| Combined | 1.7948258 | 1.4196702 | 2.2691182 |

**2. HIF-2α versus OS**
